# Supplementary material for: Influence of Organ Culture on the Characteristics of the Human Limbal Stem Cell Niche
Source: Int J Mol Sci. 2023 Nov 28;24(23):16856. doi: 10.3390/ijms242316856 (PMC10706739; doi:10.3390/ijms242316856)
Supplement: Supplementary file 1 [file ijms-24-16856-s001.zip › ijms-2737853-supplementary.pdf]

Influence of organ culture on the characteristics of the human limbal stem cell niche

**Naresh Polisetti [1,*], Gottfried Martin [1], Eva Ulrich [1], Mateusz Glegola [1],
Ursula Schlötzer-Schrehardt [2], Günther Schlunck [1] and Thomas Reinhard [1]**

Supplementary Figures:

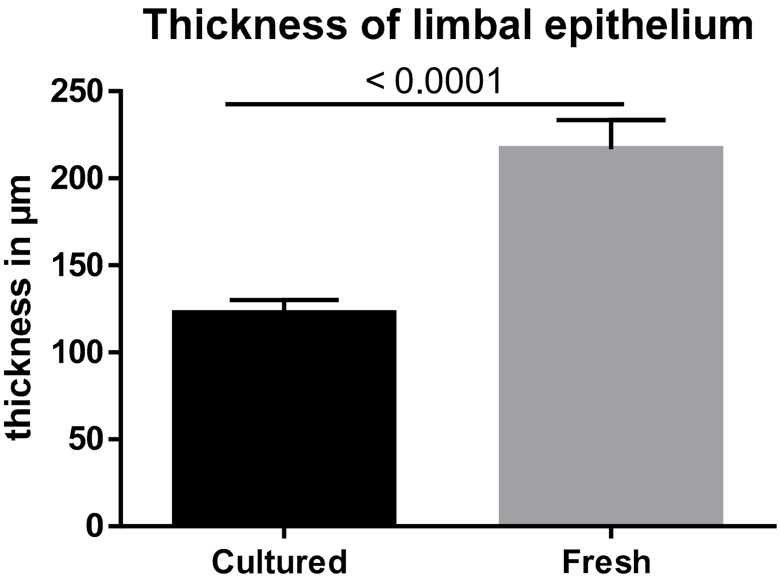

Supplementary Figure S1: The bar graph showing the thickenss of limbal epithelium of both organ-cultured (n=4) and fresh tissue sections (n=3). Data are expressed means ± standard errors of mean. ****p<0.0001; Mann-Whitney *U* test

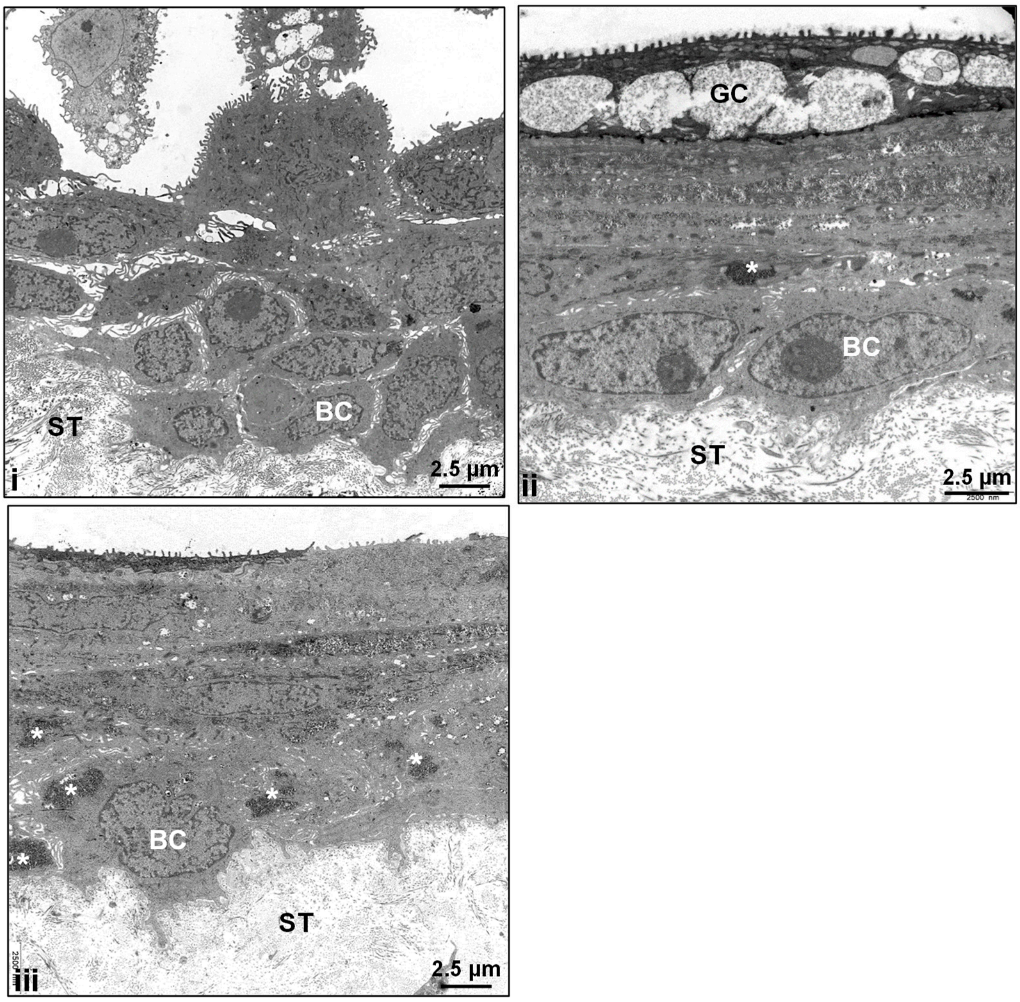

Supplementary Figure S2: Transmission electron micrographs of organ-cultured limbal tissues showing the desquamation of superficial epithelial cells and loosely connected epithelial cells (i; BC, basal cells; ST, stroma); accumulation of glycogen in both basal and superficial cells (asterisk, ii & iii); and goblet-like cells in the superficial epithelial layers (iii).

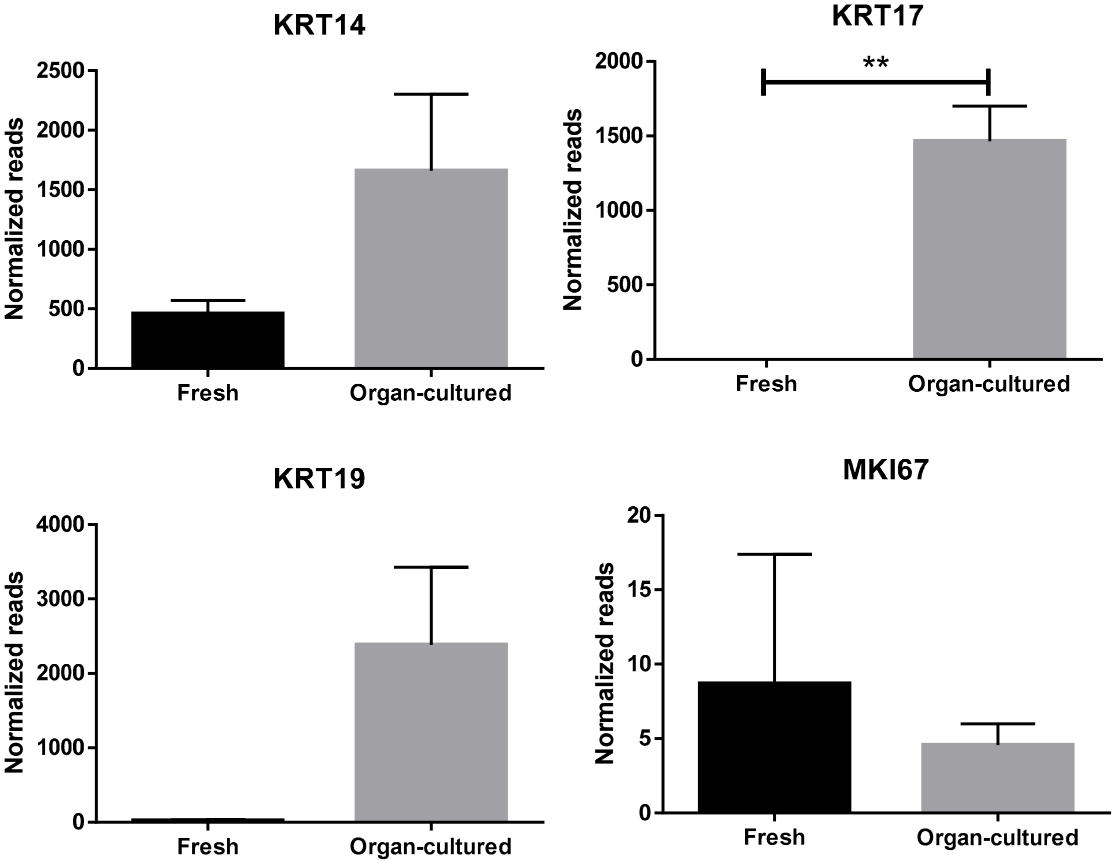

Supplementary Figure S3: RNA-sequencing data analysis of cytokeratin(CK/KRT)14, KRT17, KRT19, and Ki-67 (MKI67) expression in both fresh and organ-cultured cornea. Data are expressed means ± standard errors of the mean. **$p < 0.01$, Mann-Whitney *U* test

A

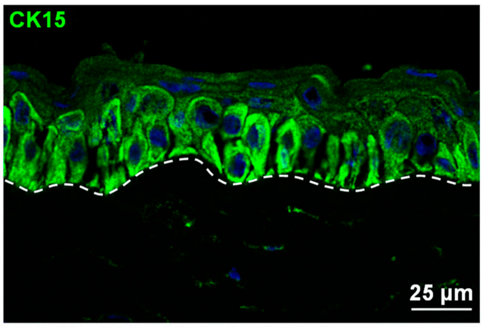

B

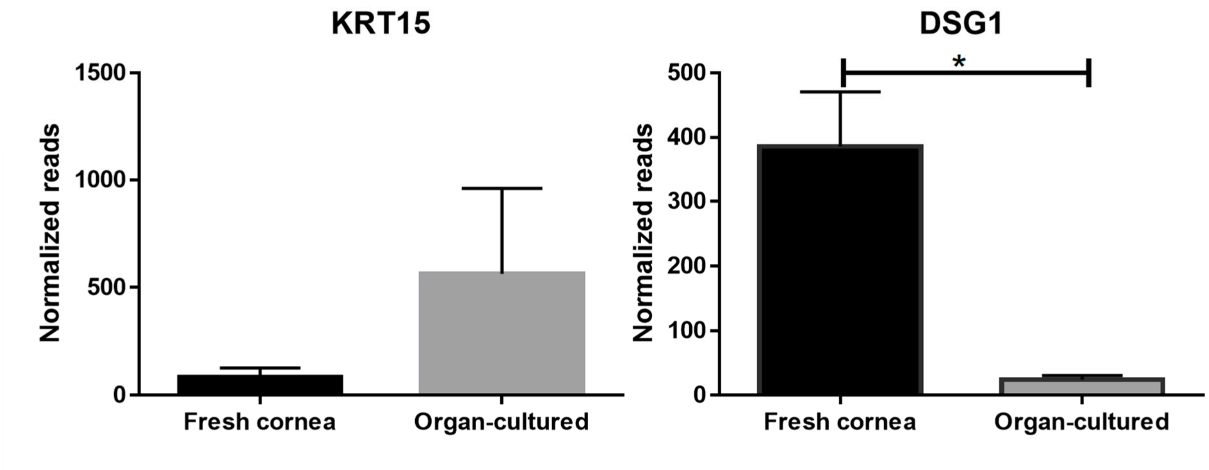

Supplementary Figure S4: A) Immunohistochemical analysis of organ-cultured limbal tissue section showing the cytokeratin (CK) 15 expression (green) in the basal cells of the peripheral cornea. The dashed line represents the basement membrane. Nuclear counterstaining with 4′,6-diamidino-2-phenylindole (blue). B) RNA-sequencing data analysis of cytokeratin (CK/KRT) 15 and desmoglein (DSG1) in both fresh and organ-cultured cornea. Data are expressed means ± standard errors of the mean. *$p < 0.05$, Mann-Whitney *U* test

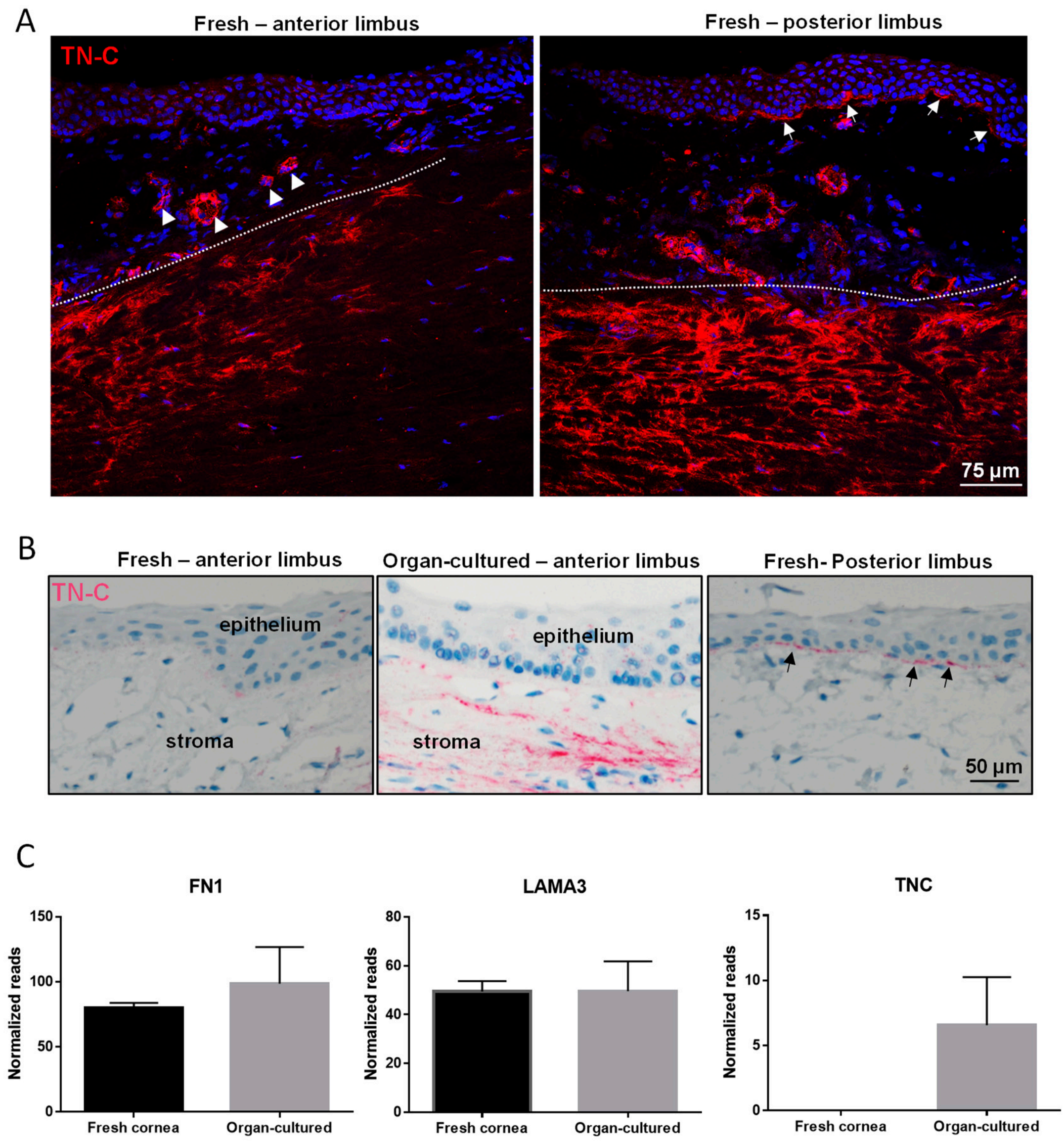

Supplementary Figure S5: A) Immunohistochemical analysis of frozen limbal tissue sections showing the Tenascin (TN)-C expression (red) in the corneoscleral interface (dotted line separates the limbus and the sclera) and vessels (arrows heads), as well as in the basement membrane of the posterior limbus (arrows). Nucelar counterstaining with 4′,6-diamidino-2-phenylindole (blue). B) Immunohistochemical analysis on paraffin sections of organ-cultured limbal tissue shows a similar pattern of expression of TN-C in both fresh and organ-cultured tissues. C) RNA-sequencing data analysis of fibronectin (FN1), laminin α3 (LAMA3) and TN-C (TNC) of both fresh and organ-cultured cornea. Data are expressed means ± standard errors of the mean.

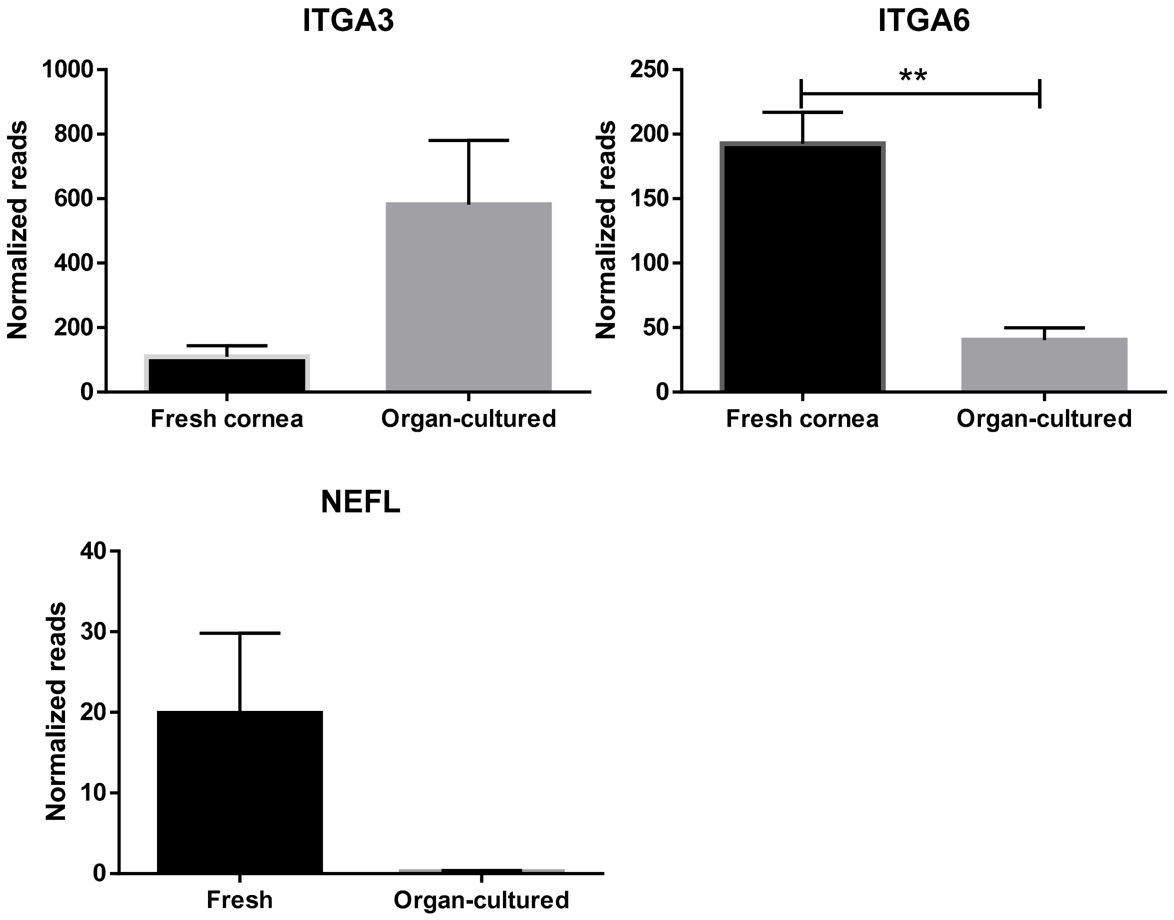

Supplementary Figure S6: RNA-sequencing data analysis of integrin $\alpha 3$ (ITGA3), ITGA6, and neurofilament (NF/NEF)L in both fresh and organ-cultured cornea. Data are expressed means ± standard errors of the mean. **$p < 0.01$, Mann-Whitney *U* test

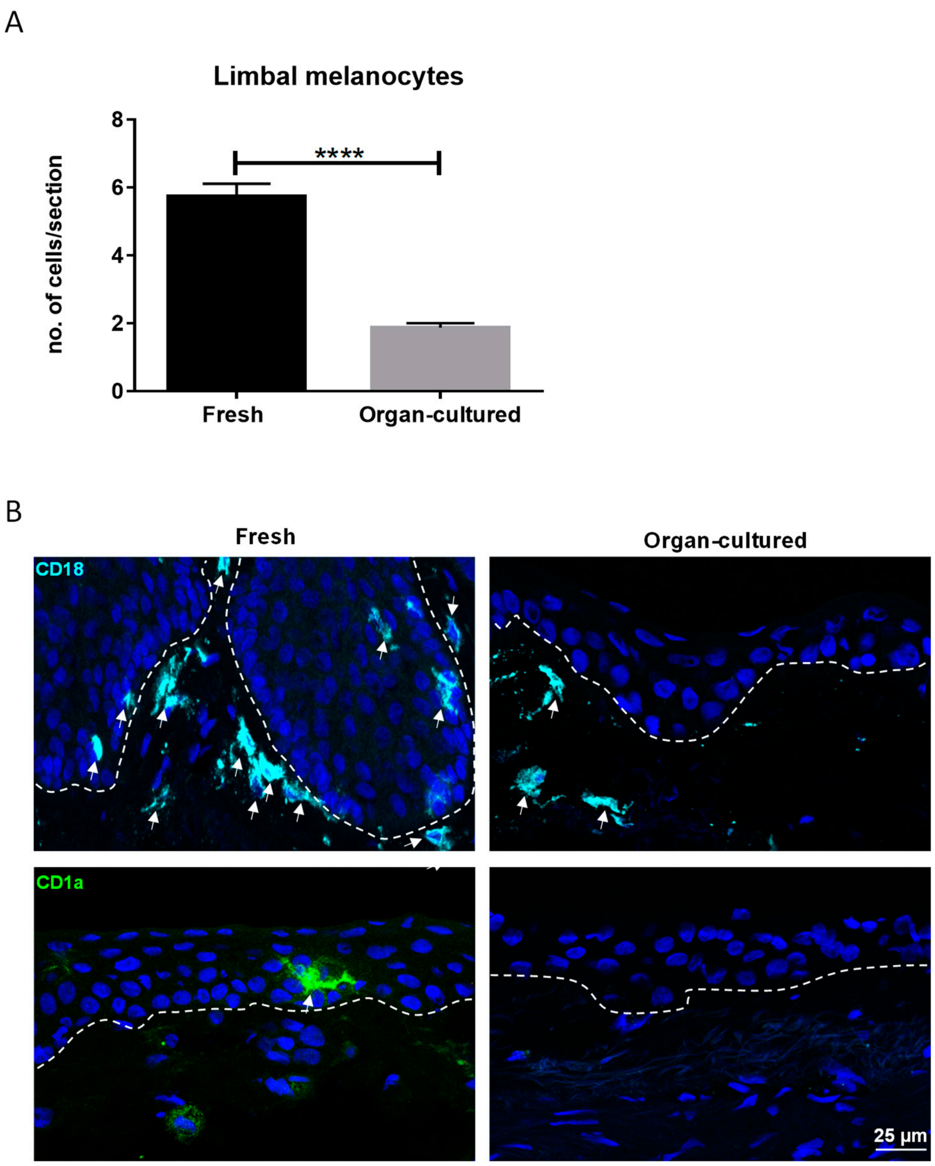

Supplementary Figure S7: A) A bar graph showing the number of melanocytes present in the fresh (n=3) and organ-cultured limbal tissue sections (n=4). Data are expressed means ± standard errors of the mean. ****$p < 0.0001$. Mann-Whitney *U* test. B) Immunohistochemical analysis of limbal tissue sections showing the CD18 (cyan, arrows) positive cells in the stroma as well as in the epithelial layers of fresh tissues, whereas positive cells are only observed in the stroma of organ-cultured tissues; CD1a positive cells (green, arrow) in the limbal epithelium of fresh tissues but not in the organ-cultured tissues. The dashed line represents the basement membrane. Nuclear counterstaining with 4′,6-diamidino-2-phenylindole (blue).

Supplementary Table

	Antigens	Supplier	Code	Clone	Host	Application	Dilution
1	CD1a	Abcam	ab238463	NA1/34	Mouse	IHC-F	1000
2	CD18	Bio-rad	MCA503GA	YFC118.3	Rat	IHC-F	1000
3	CD44	Biolegend	103023	IM7	Rat	IHC-F	200
4	CD90	BD Pharmingen	559869	5E10	Mouse	IHC-F	200
5	Cadherin, neural(N)	CST	13116S	D4R1H	Rabbit	IHC-F	100
6	Cadherin, placental (P)	R&D sytems	FAB861G	104805	Mouse	IHC-F	25
7	Collagen type IV	SouthernBiotech	1460-02	2F11	Mouse	IHC-F	600
8	β-dytroglycan	Novus	NBP3-13643	GT835	Mouse	IHC-F	500
9	Desmogelin (DSG)-1	Thermo	32-6000	27B2	Mouse	IHC-F	200
10	E-cadherin	Cell Signaling	3195	24E10	Mouse	IHC-F	600
11	Fibronectin	Sigma	F0916	IST-4	Mouse	IHC-F	500
12	HLA-DRA	Abcam	ab92511	EPR3692	Rabbit	IHC-F	1000
13	Integrin alpha 3	Sigma	MAB1952Z	P1B5	Mouse	IHC-F	300
14	Integrin alpha 6	Merck	MAB1378	NKI-GoH3	Rat	IHC-F	200
15	Ki-67	DAKO	M7240	MIB-1	Mouse	IHC-P	100
16	Cytokeratin 12	Abcam	ab185627	EPR17882	Rabbit	IHC-P	500
17	Cytokeratin 14	Abcam	ab7800	LL002	Mouse	IHC-P	200
18	Cytokeratin 15	Invitrogen	MA5-11344	LHK15	Mouse	IHC-F	500
19	Cytokeratin 17/19	CST	12434S	D4G2	Rabbit	IHC-P	200
20	Laminin α3	R&D sytems	MAB21441	546215	Mouse	IHC-F	100
21	Laminin α5	Millipore	MAB1924	4C7	Mouse	IHC-F	100
22	Melan-A	Abcam	ab210546	EPR20380	Rabbit	IHC-F	1000
23	Neurofilament	Agilent	M076229-2	2F11	Mouse	IHC-F	100
24	Tenascin -C	Abcam	ab108930	EPR4219	Rabbit	IHC-F/P	500
25	TP63	CST	67825	E6Q3O	Rabbit	IHC-P	100
26	Vimentin	CST	5741S	D21H3	Rabbit	IHC-F	500
27	von Willebrand factor	Sigma	F3520	polyclonal	Rabbit	IHC-F	1000

Supplementary Table S1: Lis of antibodies. Abbreviations: IHC, immunohistochemistry, IHC-F; IHC-frozen; IHC-P- IHC-paraffin
